# Safety, tolerability, pharmacokinetics, and efficacy of AMG 403, a human anti-nerve growth factor monoclonal antibody, in two phase I studies with healthy volunteers and knee osteoarthritis subjects

**DOI:** 10.1186/s13075-015-0797-9

**Published:** 2015-10-08

**Authors:** Jason M. Gow, Wayne H. Tsuji, Gary J. Williams, Daniel Mytych, David Sciberras, Shawn L. Searle, Tim Mant, John P. Gibbs

**Affiliations:** Amgen, Inc, 1201 Amgen Court West, Seattle, WA 98119 USA; Amgen, Inc, One Amgen Center Drive, Thousand Oaks, CA 91320 USA; Amgen Limited, 240 Cambridge Science Park, Milton Road, Cambridge, CB4 0WD UK; PRA Health Sciences, 3838 South 700 East, Salt Lake City, UT 84106 USA; Quintiles Drug Research Unit at Guy’s Hospital, 6 Newcomen Street, London, SE1 1YR UK; Quest Diagnostics, Inc, 1311 Calle Batido, San Clemente, CA 92673 USA

## Abstract

**Introduction:**

Nerve growth factor plays a key role in the pathology of osteoarthritis (OA) related chronic pain. The aim of these studies was to evaluate the safety, tolerability, pharmacokinetics, and clinical response of AMG 403, a human anti-nerve growth factor monoclonal antibody, in healthy volunteers and subjects with knee OA.

**Methods:**

Two phase I, randomized, placebo-controlled, double-blind studies were conducted. The single-ascending dose study randomized healthy volunteers (*n* = 48) 3:1 to receive AMG 403 (1, 3, 10, or 30 mg intravenously; or 10 or 30 mg subcutaneously; *n* = 8 per group) or placebo. The multiple-ascending dose study randomized knee OA subjects (*n* = 18) 3:1 to receive AMG 403 (3, 10, or 20 mg subcutaneously once monthly for four doses) or placebo. Safety, tolerability, and pharmacokinetics (PK) were assessed for both studies. Patient’s and physician’s disease assessments and total WOMAC score were determined in knee OA subjects.

**Results:**

AMG 403 appeared to be well-tolerated after single and multiple doses, except for subject-reported hyperesthesia, pain, and paresthesia (mild to moderate severity). These treatment-emergent neurosensory events showed evidence of reversibility and a possible dose-dependence. Three serious adverse events were reported in AMG 403 treated subjects, but were not considered treatment related. AMG 403 PK was linear with an estimated half-life of 19.6 to 25.8 days. After multiple doses, AMG 403 PK showed modest accumulation (≤2.4-fold increase) in systemic exposure. Knee OA diagnosis, body weight, and anti-drug antibody development did not appear to affect AMG 403 PK. Patient’s and physician’s disease assessments and total WOMAC score showed improvement in AMG 403 treated knee OA subjects compared with placebo.

**Conclusions:**

AMG 403 was generally safe and well-tolerated in both healthy volunteers and knee OA patients, and exhibited linear pharmacokinetics. Preliminary clinical efficacy was observed in knee OA subjects.

**Trial registration:**

ClinicalTrials.gov NCT02348879. Registered 23 December 2014. Clintrials.gov NCT02318407. Registered 2 December 2014.

**Electronic supplementary material:**

The online version of this article (doi:10.1186/s13075-015-0797-9) contains supplementary material, which is available to authorized users.

## Introduction

Osteoarthritis (OA) is a painful and disabling inflammatory disease of the joints and is the most prevalent form of arthritis, affecting more than 26 million US adults [1–3]. It is caused by multiple factors (e.g., joint injury or overuse, obesity, and heredity) and dramatically increases in prevalence with age [1]. The disease pathology involves breakdown of joint cartilage, tendon and ligament stretch, and when severe, bone erosion [4]. OA can affect the hands and weight-bearing joints, such as knees, hips, feet, and lower back [5]. Joint stiffness, pain, and loss of joint movement are typical symptomatic manifestations that are used to assess disease severity based on various metrics, such as the Western Ontario and McMaster Universities Osteoarthritis Index (WOMAC) [6, 7].

Pain is the principal reason why OA patients seek medical care and it is a major determinant of other OA disease outcomes such as disability and joint replacement [8, 9]. Chronic use of commonly prescribed therapies for OA pain, such as nonsteroidal anti-inflammatory drugs (NSAIDs) and opioids, is often hindered by safety issues and the development of tolerance [10]. While the etiology of OA pain is unclear, the investigation of novel pain pathways is necessary to provide improved treatment over the typical therapies, many of which were approved decades ago.

Nerve growth factor (NGF) contributes to persistent pain in a variety of animal models and is elevated in damaged peripheral tissues, and recent reviews thoroughly present the rationale for targeting NGF in treating OA pain [11, 12]. A member of the neurotrophin family, NGF is a secreted, soluble protein that binds two different cell surface receptors: the 75 kDa neurotrophin receptor (p75NTR) which binds all neurotrophins with comparable affinity, and the high-affinity NGF-specific tyrosine kinase receptor (TrkA) that shows selectivity for NGF over other neurotrophins [13]. Certain genetic variants of NGF or TrkA are linked to decreased nociception or congenital insensitivity, and elevated NGF levels are associated with many chronic pain conditions [14, 15]. Preclinical use of anti-NGF antibodies has demonstrated efficacy in rodent models of inflammatory and neuropathic pain [16]. Furthermore, sequestration of NGF via an anti-NGF antibody has shown promising pain relief for OA patients in clinical trials [17–19].

AMG 403 is a fully human immunoglobulin G2 (IgG2) monoclonal antibody that selectively binds NGF. It is anticipated that AMG 403 will provide a new treatment option for chronic OA pain, without causing sedation or dependence. AMG 403, also known as fulranumab, is currently in clinical development. We report here the safety, tolerability, pharmacokinetics (PK), and clinical effect of AMG 403 observed in two randomized, double-blind, placebo-controlled phase I studies in healthy volunteers and patients with knee OA.

## Methods

The protocol and informed consent documentation for the two phase I clinical studies were reviewed and approved by the independent ethics committee or institutional review board specific for the study center and are listed in Additional file 1. The studies were conducted in accordance with the International Conference on Harmonisation and Good Clinical Practice regulations and guidelines. Written informed consent was obtained from all study participants before any screening or study-related procedures were performed.

### Single-ascending dose study in healthy volunteers

This was a randomized, double-blind, placebo-controlled, sequential, single-ascending dose study conducted from 18 January 2005 through 12 January 2006 at a single study site. Inclusion criteria for enrollment followed the standard practice for first-in-human studies. Briefly, eligible healthy male and female volunteers were studied, who were of non-child bearing potential between 18 and 55 years of age (inclusive), with a body mass index (BMI) of 18 to 29 kg/m^2^. Clinical laboratory measurements and 12-lead electrocardiogram intervals were assessed for general health during the 21-day screening period. The day before dosing, subjects were randomized to a single dose of AMG 403 or placebo in a 3:1 ratio for each dose regimen. The double-blind treatment/follow-up period was either 49 (1 and 3 mg AMG 403) or 91 (10 and 30 mg AMG 403) days in duration. Single doses of AMG 403 were administered by either one-hour intravenous (IV) infusion (1, 3, 10, and 30 mg) or subcutaneous (SC) injection (10 and 30 mg).

### Multiple-ascending dose study in patients with knee OA

This was a randomized, double-blind, placebo-controlled, sequential, multiple-dose study conducted at three sites from 13 December 2006 through 17 January 2008. Eligible male and female subjects 18 to 65 years of age (inclusive), body weight <125 kg, and diagnosed with knee OA (as defined by the following American College of Rheumatology criteria: knee pain and radiographic evidence of osteophytes, and morning stiffness for ≤30 minutes and/or crepitus on motion) were enrolled. At screening, subjects were to have visual analog scale (VAS) pain scores ≥30 mm for the index knee and clinically acceptable laboratory tests and electrocardiogram results. If patients were taking any non-prescribed supplements or prescription medications including pain medication, the doses were to have been stable for at least 1 month.

Key exclusion criteria included active or prior history of peripheral neuropathy, paresthesia, or dysesthesia or any other previously diagnosed neurologic condition causing the above noted symptoms; uncontrolled diabetes, cardiovascular disease or hypertension; diagnosis of a condition other than knee OA that could cause or affect pain or pain assessment in the index knee (e.g., radiculopathy or neuropathy, vasculopathy, fibromyalgia or active depression); neuromodulatory agents used as analgesic therapy for neuropathic pain; planned surgical treatment for OA during the study period; inflammatory arthropathy including secondary OA; smoking more than 20 cigarettes per day within the 12 months prior to first dose administration; women of child-bearing potential; current or recent use of opiates or Diacerein (≤1 week prior to first AMG 403 dose administration).

The study design consisted of a 21-day screening, 112-day double-blind efficacy, and 126-day post-treatment follow-up period. In each dose cohort (3, 10, or 20 mg AMG 403), subjects were randomized 3:1 to receive AMG 403 or placebo, once every 4 weeks (Q4W) by SC injection for a total of four dose administrations. Administration of the second, third, and fourth doses occurred only after the previous dose was found to be safe and well-tolerated based on protocol-specified stopping rules. Dose escalation to the next dose cohort proceeded only when the previous dose cohort was found to be safe and well-tolerated following a review of all available safety data to day 22.

### Safety

Safety assessments included vital signs, laboratory tests, 12-lead electrocardiogram, and neurological and injection site evaluations for the determination of adverse events (AE) and serious adverse events (SAE) from study enrollment to end of study.

### Immunogenicity

Serum samples were collected from healthy volunteers (single-dose study) on study days 1 (pre-dose), 29, 50 (only 1, 3, and 10 mg IV), or day 92 (only 10 and 30 mg IV, and 10 and 30 mg SC), and from patients with knee OA (multiple-dose study) on study days 1 (pre-dose), 29 (pre-dose), 57 (pre-dose), 85 (pre-dose), 197 (only 3 and 10 mg SC Q4W), or 211 (20 mg SC Q4W). All samples were first tested for anti-drug antibodies (ADA) using a validated electrochemiluminescent (ECL) immunoassay to screen and confirm antibodies capable of binding AMG 403. Samples testing positive in the ECL immunoassay were subsequently tested in a validated cell-based bioassay to detect AMG 403 neutralizing or inhibitory effects in vitro. Samples that tested negative in the ECL immunoassay were not tested in the bioassay. Subjects were designated with a positive immune response if antibodies to AMG 403 were detected at any time point subsequent to the first dose administration.

### Pharmacokinetics

Serum samples were collected from subjects while on study at protocol-specific time points. Serum AMG 403 levels were measured using validated enzyme-linked immunosorbent assays with a lower limit of quantification (LLOQ) of 0.0016 or 0.0051 μg/mL for the single- and multiple-dose studies, respectively. PK parameters were derived from non-compartmental analysis using Phoenix WinNonlin 6.3 (Pharsight, Mountain View, CA, USA).

### Population PK modeling with covariate analysis

From model development a two-compartment model was identified as the appropriate choice for a combined analysis of the individual PK data from healthy volunteers and patients with knee OA, using NONMEM 7.2 (ICON Development Solutions, Ellicott City, MD, USA). The following population PK (pop PK) parameters were estimated: first-order SC absorption rate (k_a_), clearance (CL), central volume of distribution (V_c_), peripheral volume of distribution (V_p_), intercompartmental CL (Q), and bioavailability (F) of a SC dose. Baseline body weight was evaluated as a continuous covariate on CL and V_c_. Knee OA diagnosis and ADA development to AMG 403 were independently tested as dichotomous covariates on CL.

### Efficacy in patients with knee OA

A 100-mm VAS was used for patients’ self-assessment of knee OA as “very good” to “very poor” [20]. Physician disease assessment rated the condition of the study subject’s knee on a scale of 0 to 4 (0 = very good and 4 = very poor). Study subjects independently reported answers to the Western Ontario and McMaster Universities Osteoarthritis Index (WOMAC) 3.1 questionnaire to measure the three components of OA symptoms: pain, stiffness, and physical function. Baseline determinations for all three efficacy endpoints were assessed prior to the first dose administration and at various time points during treatment.

### Statistical analysis

All subjects who received at least one dose of AMG 403 were included in the safety and efficacy analyses. Adverse events, PK, and efficacy endpoints were descriptively compared across the various treatment groups, as the study was not powered to perform formal statistical testing.

## Results

### Study subject characteristics

Of the 46 healthy volunteer subjects enrolled in the single-dose study, 34 received AMG 403 and 12 received matching placebo. In the multiple-dose knee OA study, 18 subjects received AMG 403 and six subjects received matching placebo (Table 1). Mean age and weight were typically higher for patients with knee OA than for healthy volunteer subjects. For both studies combined, all subjects were Caucasian, except for three African-Americans in the multiple-dose study (one each in the placebo, 10-mg, and 20-mg SC Q4W groups), and most subjects were male (77 %). Disease activity at screening in patients with knee OA in the multiple-dose study was generally similar across the cohorts.

**Table 1:** Subject demographics and disease characteristics

	Single ascending dose^a^							Multiple ascending dose^a^			
Demographics and disease characteristics		IV				SC		SC Q4W × 4			
	Placebo^b^	1 mg	3 mg	10 mg	30 mg	10 mg	30 mg	Placebo	3 mg	10 mg	20 mg
	(N = 12)	(N = 6)	(N = 6)	(N = 5)	(N = 6)	(N = 5)	(N = 6)	(N = 6)	(N = 6)	(N = 6)	(N = 6)
Age, years	25.2	35.0	30.2	22.8	46.7	31.6	28.2	54.7	53.0	48.7	52.7
	(6.3)	(12.0)	(11.2)	(2.8)	(5.0)	(9.2)	(9.5)	(9.7)	(6.8)	(8.7)	(5.5)
Weight, kg	73.1	73.8	76.8	71.2	74.2	82.8	76.7	81.5	93.4	101	112
	(91)	(6.5)	(7.8)	(12.2)	(10.0)	(8.9)	(10.7)	(6.1)	(18.1)	(16.5)	(10.3)
Male, %	92	100	100	100	83	100	100	17	67	50	50
Mean total WOMAC score	na^c^	na	na	na	na	na	na	51.1	46.3	49.8	68.7
								(9.3)	(6.4)	(8.1)	(3.7)
Mean VAS score of patients’ disease assessments	na	na	na	na	na	na	na	35.5	52.3	48.3	59.0
								(8.3)	(8.0)	(6.6)	(6.0)
Mean score of physician’s disease assessments	na	na	na	na	na	Na	na	2.17	2.33	2.50	2.00
								(0.48)	(0.33)	(0.22)	(0.26)

All data are mean (SD), unless stated otherwise. ^a^Healthy volunteers and patients with knee osteoarthritis were administered single- or multiple-doses of AMG 403, respectively. ^b^Combined intravenous (*IV*) and subcutaneous (*SC*) placebo subjects. ^c^Not applicable for healthy volunteer subjects. *Q4W* once every four weeks, *WOMAC* Western Ontario and McMaster Universities Osteoarthritis Index, *VAS* visual analog scale, *na* not applicable

### Safety

In the single-dose study with healthy volunteers, no subjects were removed from the study due to AEs and there were no deaths during the study. Only one SAE was reported (pneumothorax, 10 mg IV AMG 403), but it was not considered to be treatment-related (Table 2). Treatment-emergent adverse events (TEAEs) related to sensory symptomatology reported by 5 % or more of all subjects who received AMG 403 were headache (6/34 subjects, 18 %), pain in an extremity (4/34, 12 %), and hyperesthesia (3/34, 9 %). Furthermore, pain in the extremities and hyperesthesia were only reported at the highest dose level (30 mg IV and SC). TEAEs reported by 5 % or more of all healthy volunteers who received AMG 403 were herpes simplex (5/34 subjects, 15 %) and dizziness (3/34, 9 %). Herpes simplex/oral herpes were reported by one of 12 (8 %) placebo subjects and by one subject in each of the AMG 403 treatment groups, except 10 mg SC. The clinical significance and relationship of the reported cold sores to AMG 403 were unclear. Only one AMG 403-treated subject experienced an injection-site reaction (mild transient pruritus, 30 mg SC).

**Table 2:** Adverse events

		Single ascending dose^a^								Multiple ascending dose^a^					
			IV				SC		All	SC Q4W × 4				All	Total
Adverse events		Placebo	1 mg	3 mg	10 mg	30 mg	10 mg	30 mg	AMG 403	Placebo	3 mg	10 mg	20 mg	AMG 403	AMG 403
		(N = 12)	(N = 6)	(N = 6)	(N = 5)	(N = 6)	(N = 5)	(N = 6)	(N = 34)	(N = 6)	(N = 6)	(N = 6)	(N = 6)	(N = 18)	(N = 52)
Serious adverse events, n (%)^b^		0	0	0	1	0	0	0	1	0	0	1	1	2	3
		(0)	(0)	(0)	(20)	(0)	(0)	(0)	(3)	(0)	(0)	(17)	(17)	(11)	(6)
TEAEs reported by ≥5 % of subjects, n (%)^c^		8	3	1	5	4	3	6	22	6	6	6	6	18	40
		(67)	(50)	(17)	(100)	(67)	(60)	(100)	(64)	(100)	(100)	(100)	(100)	(100)	(77)
	Headache	3	1	0	1	1	0	3	6	2	2	1	1	4	10
		(25)	(17)	(0)	(20)	(17)	(0)	(50)	(18)	(33)	(33)	(17)	(17)	(22)	(19)
	Herpes simplex/oral herpes	1	1	1	1	1	0	1	5	0	0	0	2	2	7
		(8)	(17)	(17)	(20)	(17)	(0)	(17)	(15)	(0)	(0)	(0)	(33)	(11)	(13)
	Nasopharyngitis	2	1	0	0	1	1	2	5	0	0	1	1	2	7
		(17)	(17)	(0)	(0)	(17)	(20)	(33)	(15)	(0)	(0)	(17)	(17)	(11)	(13)
	Arthralgia	0	0	0	0	0	0	0	0	3	2	1	1	4	4
		(0)	(0)	(0)	(0)	(0)	(0)	(0)	(0)	(50)	(33)	(17)	(17)	(22)	(8)
	Dizziness	2	0	0	0	1	1	1	3	1	1	0	0	1	4
		(17)	(0)	(0)	(0)	(17)	(20)	(17)	(9)	(17)	(17)	(0)	(0)	(6)	(8)
	Pain in extremity	0	0	0	0	1	0	3	4	0	0	0	0	0	4
		(0)	(0)	(0)	(0)	(17)	(0)	(50)	(12)	(0)	(0)	(0)	(0)	(0)	(8)
	Paresthesia	1	0	0	0	1	0	0	1	0	0	0	3	3	4
		(8)	(0)	(0)	(0)	(17)	(0)	(0)	(3)	(0)	(0)	(0)	(50)	(17)	(8)
	Hyperesthesia	0	0	0	0	0	0	3	3	0	0	0	0	0	3
		(0)	(0)	(0)	(0)	(0)	(0)	(50)	(9)	(0)	(0)	(0)	(0)	(0)	(6)
	Joint effusion	0	0	0	0	0	0	0	0	0	0	1	2	3	3
		(0)	(0)	(0)	(0)	(0)	(0)	(0)	(0)	(0)	(0)	(17)	(33)	(17)	(6)
	Myalgia	1	0	0	0	1	0	0	1	1	2	0	0	2	3
		(8)	(0)	(0)	(0)	(17)	(0)	(0)	(3)	(17)	(33)	(0)	(0)	(11)	(6)
	Nausea	0	0	0	0	0	0	0	0	0	2	0	1	3	3
		(0)	(0)	(0)	(0)	(0)	(0)	(0)	(0)	(0)	(33)	(0)	(17)	(17)	(6)
	Pharyngolaryngeal pain	2	1	0	0	1	1	0	3	0	0	0	0	0	3
		(17)	(17)	(0)	(0)	(17)	(20)	(0)	(9)	(0)	(0)	(0)	(0)	(0)	(6)
	Osteoarthritis	0	0	0	0	0	0	0	0	0	2	1	0	3	3
		(0)	(0)	(0)	(0)	(0)	(0)	(0)	(0)	(0)	(33)	(17)	(0)	(17)	(6)
	Upper respiratory tract infection	0	0	0	0	0	0	0	0	4	2	1	0	3	3
		(0)	(0)	(0)	(0)	(0)	(0)	(0)	(0)	(67)	(33)	(17)	(0)	(17)	(6)
	Fatigue	0	0	0	0	1	1	0	2	0	0	0	0	0	2
		(0)	(0)	(0)	(0)	(17)	(20)	(0)	(6)	(0)	(0)	(0)	(0)	(0)	(4)
	Diarrhea	0	0	0	0	0	0	0	0	2	1	0	0	1	1
		(0)	(0)	(0)	(0)	(0)	(0)	(0)	(0)	(33)	(17)	(0)	(0)	(6)	(2)
	Back pain	2	0	0	0	0	0	0	0	0	0	0	0	0	0
		(17)	(0)	(0)	(0)	(0)	(0)	(0)	(0)	(0)	(0)	(0)	(0)	(0)	(0)
	Toothache	0	0	0	0	0	0	0	0	2	0	0	0	0	0
		(0)	(0)	(0)	(0)	(0)	(0)	(0)	(0)	(33)	(0)	(0)	(0)	(0)	(0)

^a^Healthy volunteers and patients with knee osteoarthritis were administered single- or multiple-doses of AMG 403, respectively. ^b^No serious adverse events were considered to be treatment-related. ^c^Based on placebo or AMG 403 groups combined, respectively, and ordered by event frequency for AMG 403. *IV* intravenous, *SC* subcutaneous, *TEAEs* treatment-emergent adverse events

In the multiple-dose study with patients with knee OA, AMG 403 appeared to be generally well-tolerated (Table 2) except for sensory symptomatology TEAEs: arthralgia (4/18 subjects, 22 %), headache (4/18, 22 %), paresthesia (3/18, 17 %), and myalgia (2/18, 11 %). Paresthesia TEAEs were only in the highest multiple-dose group (20 mg SC Q4W). No subjects were discontinued from the study early because of AEs and there were no deaths. Two SAEs were reported for AMG-403-treated subjects: gastrointestinal perforation (10 mg SC Q4W) and knee arthroplasty (20 mg SC Q4W). Neither SAE was considered by the investigator to be treatment-related. Two patients with knee OA in the 20-mg SC Q4W group experienced CTCAE (common terminology criteria for adverse events) grade 2 neurosensory AEs (moderate severity), which triggered the activation of a protocol-specified stopping rule for the entire dose group. As a result, three subjects on 20 mg SC Q4W who were randomized to AMG 403 did not receive the fourth dose.

The common terminology criteria for adverse events (i.e., mild, moderate, or severe) were used to characterize AEs following AMG 403 or placebo. As noted in Table 2, the pain-related AEs reported in the two studies were headache, arthralgia, myalgia, pain in extremities, and back pain. Of these AEs, 21 occurred in AMG 403-treated subjects and 12 in placebo-treated subjects. All pain-related AEs in AMG-403-treated subjects were mild to moderate in severity. It should be noted that the two pain-related AEs (arthralgia and myalgia) observed in subjects with OA could be OA-related and therefore confounding with respect to study drug use.

### Immunogenicity

A total of 14 out of 52 AMG 403-treated subjects (27 %, combined single- and multiple-dose studies) were considered ADA positive because in at least one sample there was positive binding of ADA to AMG 403 during the treatment period. In the single- and multiple-dose studies there were one (2.9 %) and 13 (72 %) of subjects who were positive for ADA binding, respectively (30 mg IV single dose (1/6 subjects); 3 mg SC Q4W (4/6), 10 mg SC Q4W (4/6), and 20 mg SC Q4W (5/6) multiple dose). None of the ADA-binding-positive samples were positive for neutralizing antibodies.

### Pharmacokinetics

**Fig. 1:**
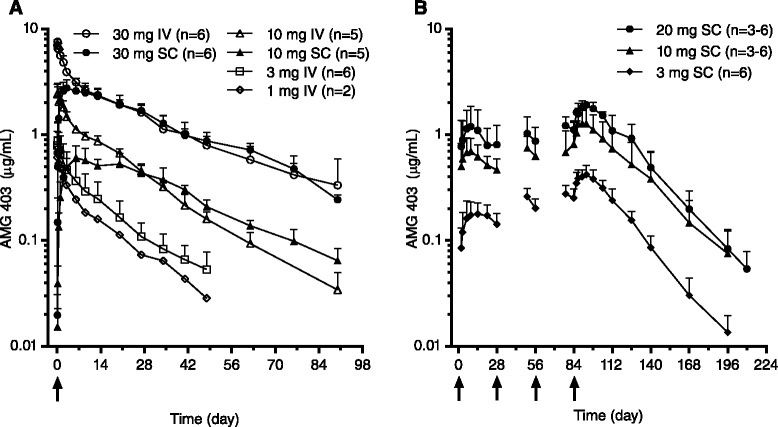
AMG 403 concentration-time profiles following single and repeat dose administration. AMG 403 was administered intravenously (*IV*) or subcutaneously (*SC*) and serum AMG 403 levels were measured pre-dose through end of study. Each point represents the mean (+SD) AMG 403 concentration. **a** Healthy volunteers received a single IV or SC dose of AMG 403 (1–30 mg, LLOQ = 0.0016 μg/mL). **b** Patients with knee osteoarthritis received a once-monthly SC dose of AMG 403 (3–20 mg, LLOQ = 0.0051 μg/mL) for up to four dose administrations. *Arrows* represent dose administration

Mean serum AMG 403 concentration-time profiles after single (healthy volunteer) and multiple (knee OA) doses are shown in Fig. 1. Four of six healthy volunteers administered a single 1-mg IV dose had serum concentrations below or near the LLOQ and were not included in the analysis. After a single AMG 403 dose, maximum serum concentrations (C_max_) and area under the concentration-time curve (AUC_0-*t*_) increased approximately dose-proportionally for IV (3 to 30 mg) and SC (10 to 30 mg) administration. Elimination rates were similar for all groups based on mean clearance (CL 0.232 to 0.329 L/day) and half-life (19.6 to 25.8 days) values. Mean SC bioavailability (F) ranged from 77.0 % to 88.5 %.

Multiple SC doses (3, 10, or 20 mg Q4W) in patients with knee OA resulted in approximately dose-proportional mean C_max_ and AUC_0-*t*_ values, and median time to C_max_ was 7.5 to 9.0 days after the fourth dose. Elimination rates (mean CL/F and half-life) were similar among the three SC Q4W dose groups. AMG 403 concentrations after the fourth dose appeared to achieve steady state and moderate accumulation was observed compared with the first dose (1.8-fold to 2.4-fold AUC_0-*t*_ increase). Collectively, both single- and multiple-dose studies indicate AMG 403 exhibits linear PK in the tested dose range.

### Population pharmacokinetic modeling with exploratory covariate analysis

**Fig. 2:**
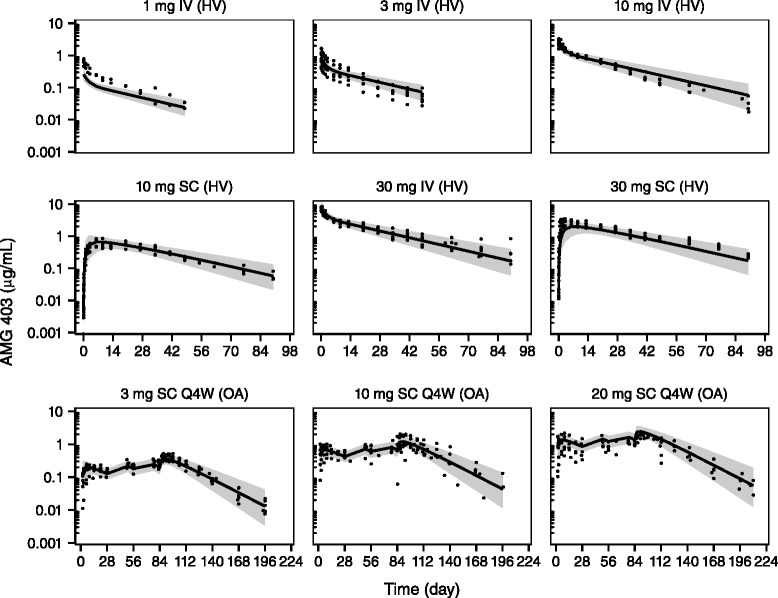
Visual predictive check of the AMG 403 population pharmokinetics (PK) model. A two-compartment model was fit simultaneously to individual AMG 403 PK data from healthy volunteers (*HV*) and patients with knee osteoarthritis (*OA*). The model parameter estimates were used to simulate 1000 trials for a visual predictive check. The predicted median PK (solid line) and 80 % prediction interval (*shaded area*) are shown with the observed individual concentrations (*solid dot*). Note the different *x-axis scales* for the single-dose and multiple-dose regimens. *IV* intravenous, *SC* subcutaneous

The base pop PK model was established by fitting a two-compartment model to the combined individual PK data from the single- and multiple-dose studies. The pop PK parameters (k_a_ = 0.175 day^−1^; CL = 0.254 L/day; V_c_ = 3.89 L; V_p_ = 2.92 L; Q = 0.784 L/day; F = 73.6 %) were estimated with good precision (standard error ≤17 %). The between-subject variability (BSV) for each parameter was 31 % to 34 %, except for k_a_ (82 %). One thousand trial simulations were performed based on the pop PK model to compare the predictions with the observed PK in a visual predictive check (VPC, Fig. 2). The results show that the majority of observed AMG 403 concentrations are within the 80 % prediction interval, indicating the observed AMG 403 PK profile and BSV are well-characterized by the pop PK model.

**Fig. 3:**
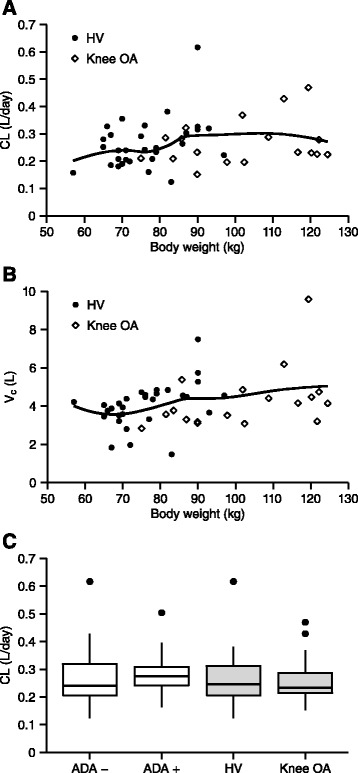
Covariate effects on AMG 403 clearance (*CL*) and (*V*_*c*_). Possible covariate effects were explored for individual CL and V_c_ parameters derived from simultaneous population pharmacokinetics modeling of data from healthy volunteers (*HV*) and patients with knee osteoarthritis (*OA*). Effect of body weight on CL (**a**) and V_c_ (**b**) is illustrated with a loess regression line. **c** The effect of anti-drug antibody (*ADA*) status (negative (n = 34) or positive (n = 14)) or disease status (HV (n = 30) or patients with knee OA (n = 18)) on CL is presented in *box plots* (median, 25th, and 75th percentiles; *whiskers* are 1.5 times the inner-quartile range and *dots* are outliers)

Select covariates that commonly influence antibody CL and V_c_, such as body weight, disease status, and ADA, were chosen for an exploratory analysis using the pop PK model (Fig. 3). Body weight (across a range of 57.0 to 124.5 kg) as a continuous covariate on either CL or V_c_ did not significantly improve the model fit. As a dichotomous covariate on CL, disease status (diagnosis of knee OA) did not affect CL nor improve the model fit. Binding ADA as a dichotomous covariate did not significantly alter CL in the pop PK model for the 14 ADA positive subjects (n = 1, single dose; n = 13, multiple dose). Median CL for ADA positive subjects compared to ADA negative subjects was negligibly higher (12 %).

### AMG 403 efficacy in patients with knee OA

**Fig. 4:**
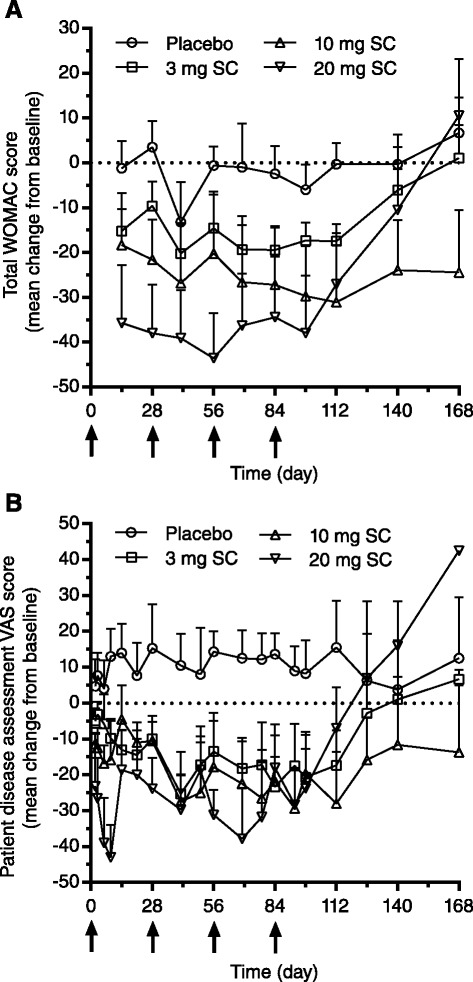
Clinical effect of AMG 403 in patients with knee osteoarthritis. Mean change (+ standard error) from baseline values over time for each dose group were plotted for total Western Ontario and McMaster Universities Osteoarthritis Index (*WOMAC*) (**a**) score and visual analog score (*VAS*) (**b**) for patient disease assessment. A*rrows* represent subcutaneous dose administrations (*SC*)

Mean change from baseline for total WOMAC scores showed an apparent dose-dependent improvement at the first time point after the first dose in the AMG 403 treatment groups and was generally maintained throughout the dosing period (Fig. 4). Marked and meaningful improvement was observed in mean change from baseline in VAS scores of patient disease assessment from the first time point after the first dose and was generally maintained throughout the dosing period. These observations were not as apparent in the placebo group. Additionally, mean change from baseline for total WOMAC scores and VAS scores for patient disease assessment showed sustained clinical effect in the AMG-403-treated groups at least 28 days after the final dose compared with the placebo group. Treatment effects for the 20 mg SC Q4W AMG 403 group trended towards baseline earlier than others after day 98, possibly because three of the six subjects in this group did not receive the fourth (final) dose of AMG 403. Results from the physician disease assessment showed a similar though less dramatic trend to the total WOMAC and patient disease assessment (data not shown).

## Discussion

NGF is a secreted factor that controls the sensitivity of primary sensory neurons and is thought to play a significant role in nociception [11, 12]. Sequestration of NGF in animal models and humans has provided compelling evidence for the use of an anti-NGF therapy in producing pain relief for OA [11, 12, 14–19]. AMG 403 is a fully human anti-NGF monoclonal antibody (IgG2) undergoing clinical development for the treatment of OA-related pain, and the current report presents safety, tolerability, pharmacokinetics, immunogenicity, and efficacy from two phase I studies with healthy volunteers and patients with knee OA.

The combined single- and multiple-dose studies showed AMG 403 was generally well-tolerated with an acceptable safety profile in both healthy volunteers and patients with knee OA. No clinically significant abnormalities in electrocardiograms, safety laboratory tests, or vital signs were reported. The three SAEs were not considered treatment-related. The neurosensory TEAEs were likely related to AMG 403 sequestration of NGF. The CTCAE grade 2 neurosensory AEs experienced by two patients with knee OA in the 20 mg SC Q4W group resolved over time, but activated a protocol-specified cohort stopping rule that prevented administration of the fourth AMG 403 dose to three subjects. The majority of TEAEs did not appear to be dose-dependent, however, paresthesia, pain in an extremity, and hyperesthesia were only observed at the highest dose level (30 mg AMG 403) for AMG-403-treated subjects and showed reversibility. A phase II study of AMG 403 (fulranumab) in patients with knee or hip OA (n = 476) also reported paresthesia as a common TEAE (7 %) in AMG-403-treated patients but a dose-dependent effect was not observed [21]. A meta-analysis of patients with knee or hip OA from 13 clinical trials of the three anti-NGF antibody therapies (tanezumab, n = 10; fasinumab, n = 1; AMG 403, n = 2) assessed the effects of potentially dose-limiting AEs from anti-NGF treatment based on a patient’s ability to remain on study. It was reported that higher anti-NGF antibody doses had a higher odds ratio for the rate of subject withdrawal due to AEs compared with placebo [22]. In the same analysis, however, lower doses were comparable with placebo for rate of subject withdrawal.

AMG 403 immunogenicity was limited to ADA-positive responses in binding assays that were all negative for neutralizing antibodies. The greater ADA incidence in the multiple-dose study (72 %, patients with knee OA) compared with the single-dose study (2.8 %, healthy volunteers) will need further investigation. A lower ADA incidence (<2 %) was observed in phase II studies with AMG 403 using revised immunogenicity assay methodology to reduce the potential for false-positive ADA caused by NGF dimer interference [23]. It is possible the immunogenicity assay methodology for the current studies could have yielded greater false-positive ADA results.

AMG 403 PK was linear and approximately dose-proportional, and SC administration showed substantial bioavailability. Integration of the single- and multiple-dose PK data into a pop PK model established a predictive simulation tool that effectively recapitulated the observed data and provided a foundation for integrating subsequent clinical PK data for identifying novel dose levels and/or regimens. Additionally, the pop PK model enabled an exploratory covariate analysis to quantify the effects of mechanistically plausible covariates (body weight, ADA, and disease status) on CL and/or V_c_. While it is common for bodyweight to influence the CL or V_c_ of monoclonal antibodies [24], it was not found to be a covariate for AMG 403. It is possible a larger population with a greater range of body weight is needed to better characterize the impact of body weight on AMG 403 PK. ADA did not appear to alter the elimination of AMG 403 since ADA was not a covariate on CL in the current analysis. Finally, NGF levels were not measured in healthy volunteers and patients with knee OA; however, disease status was not a PK covariate on CL suggesting OA-dependent effects on NGF levels [14, 15] are not expected to significantly alter AMG 403 PK.

Patients with knee OA treated with AMG 403 showed signs of clinical benefit compared with placebo-treated subjects after multiple doses. Mean total WOMAC scores decreased from baseline values at the first time point (study day 15) to beyond the fourth and final dose (study day 98) in an apparent dose-dependent manner. Additionally, patients’ disease assessments decreased markedly from baseline during the same period. Clinical effect based on physician disease assessment was less striking but generally showed a trend for improvement compared with placebo. Although variability of the efficacy data was notable and the study was not powered to evaluate the statistical significance of the efficacy measures, reported and observed pain-related treatment effects seemed apparent for AMG 403 with sustained response beyond the final dose. An adequately powered, subsequent phase II study with AMG 403 established clinically significant efficacy compared with placebo using similar pain measures to those used in the current report [21].

## Conclusions

Blockade of NGF is a recent therapeutic paradigm for treating chronic pain due to OA and may provide an improved alternative for OA patients or possibly patients with chronic neuropathic pain due to other diseases. These phase I studies showed AMG 403, a human anti-NGF monoclonal antibody, was generally safe, well-tolerated, and exhibited linear, dose-proportional PK. Intrinsic factors such as body weight, ADA, or disease status did not appear to influence AMG 403 PK, but future study will refine these characterizations. Initial clinical efficacy results support the ongoing development of AMG 403 (now fulranumab) for the treatment of knee and hip OA.

## Additional file

Additional file 1:
**Ethical review boards that provided approval for this study.** (DOC 22 kb)

## Abbreviations

ADA: anti-drug antibody
AE: adverse events
AUC_0-*t*_: area under the concentration-time curve
BMI: body mass index
BSV: between-subject variability
CL: clearance
C_max_: maximum serum concentration
ECL: electrochemiluminescent
F: bioavailability
IgG2: immunoglobulin G2
IV: intravenous
k_a_: SC absorption rate
LLOQ: lower limit of quantification
NGF: nerve growth factor
NSAID: nonsteroidal anti-inflammatory drugs
OA: osteoarthritis
p75NTR: 75 kDa neurotrophin receptor
PK: pharmacokinetics
Pop PK: population pharmacokinetics
Q: intercompartmental CL
Q4W: once every four weeks
SAE: serious adverse event
SC: subcutaneous
TEAE: treatment-emergent adverse event
TrkA: tyrosine kinase A
VAS: visual analog scale
V_c_: central volume of distribution
V_p_: peripheral volume of distribution
WOMAC: Western Ontario and McMaster Universities Osteoarthritis Index
