# Supplementary material for: Safety, tolerability, pharmacokinetics, and efficacy of AMG 403, a human anti-nerve growth factor monoclonal antibody, in two phase I studies with healthy volunteers and knee osteoarthritis subjects
Source: Arthritis Res Ther. 2015 Oct 8;17:282. doi: 10.1186/s13075-015-0797-9 (PMC4599327; doi:10.1186/s13075-015-0797-9)
Supplement: Additional file 1: — Ethical review boards that provided approval for this study. (DOC 22 kb) [file 13075_2015_797_MOESM1_ESM.doc]

**Additional file 1**

Ethical review boards that provided approval for this study are listed below:

- Independent Research Ethics Committee C/O Miss Jo Kaiserman, Faculty of Humanities, Devonshire House
- Guy’s Research Ethics Committee
- Liberty IRB
